# Associations of Socio-Demographic, Clinical and Biochemical Parameters with Healthcare Cost, Health- and Renal-Related Quality of Life in Hemodialysis Patients: A Clinical Observational Study

**DOI:** 10.3390/ijerph17186552

**Published:** 2020-09-09

**Authors:** Khanh Vuong Diem Doan, Hien Thi Minh Nguyen, Nhi Thi Hong Nguyen, Khoa Cao Dang, Shwu-Huey Yang, Tuyen Van Duong

**Affiliations:** 1 Faculty of Public Health, University of Medicine and Pharmacy, Hue University, Thua Thien Hue 491-20, Vietnam; dvdkhanh@hueuni.edu.vn (K.V.D.D.); dckhoa@hueuni.edu.vn (K.C.D.); 2 Institute for Community Health Research, University of Medicine and Pharmacy, Hue University, Thua Thien Hue 491-20, Vietnam; 3 Hemodialysis Department, Quang Ngai Provincial General Hospital, Quang Ngai 531-14, Vietnam; hienmd96@gmail.com; 4 Health Management Training Institute, University of Medicine and Pharmacy, Hue University, Thua Thien Hue 491-20, Vietnam; nthnhi@hueuni.edu.vn; 5 School of Nutrition and Health Sciences, Taipei Medical University, Taipei 110-31, Taiwan; sherry@tmu.edu.tw; 6 Research Center of Geriatric Nutrition, Taipei Medical University, Taipei 110-31, Taiwan; 7 Nutrition Research Center, Taipei Medical University Hospital, Taipei 110-31, Taiwan

**Keywords:** end-stage renal disease, hemodialysis, comorbidity, malnutrition, anemia, healthcare cost, health-related quality of life, kidney disease quality of life, low-resourced setting, Vietnam

## Abstract

We examined factors associated with healthcare cost, health-related quality of life (HRQOL), and kidney disease quality of life (KDQOL) in hemodialysis patients. We conducted a cross-sectional study on 160 patients from January to April 2019 at a hemodialysis center. Socio-demographic, clinical, and laboratory parameters and quality of life (QOL) (using KDQOL-SF-v1.3) were assessed. Monthly healthcare costs were extracted from the hospital information system. The means of healthcare cost, HRQOL, and KDQOL were VND 9.4 ± 1.6 million, VND 45.1 ± 21.9 and VND 51.3 ± 13.0, respectively. In the multivariate analysis, the healthcare cost was higher in patients with a longer hemodialysis vintage (regression coefficient (B): 0.74; 95% confidence interval (95% CI): 0.25; 1.23), comorbidity (B: 0.77; 95% CI: 0.24; 1.31); and lower in those with a higher hematocrit concentration (B: −0.07; 95% CI: −0.13; −0.01). Patients that lived in urban areas (B: 9.08; 95% CI: 2.30; 15.85) had a better HRQOL; those with a comorbidity (B: −14.20; 95% CI: −21.43; −6.97), and with hypoalbuminemia (B: −9.31; 95% CI: −16.58; −2.04) had a poorer HRQOL. Patients with a higher level of education (B: 5.38~6.29) had a better KDQOL; those with a comorbidity had a poorer KDQOL (B: −6.17; 95% CI: −10.49; −1.85). In conclusion, a longer hemodialysis vintage, a comorbidity and a lower hematocrit concentration were associated with higher healthcare costs. Patients who lived in urban areas had a better HRQOL and a higher level of education led to a better KDQOL. Patients with a comorbidity had a lower HRQOL and KDQOL. Malnourished patients had a lower HRQOL.

## 1. Introduction

The prevalence and incidence of end-stage renal disease (ESRD) have been increasing around the globe [1]. The disease has created a huge economic burden on patients and the healthcare system [2,3,4]. In Vietnam, a registration system is not available for chronic kidney disease (CKD) and ESRD. According to a previous study in 2018, three available renal replacement therapies are hemodialysis, peritoneal dialysis, and renal transplantation. Among those, hemodialysis is the most common modality, with about 15,000 patients [5]. In Vietnam, the healthcare system can provide treatments for around 10% of ESRD patients, and around 10% of patients who required hemodialysis therapy received the treatment [6].

Patients receiving hospital-based hemodialysis pay higher healthcare and societal costs than peritoneal dialysis patients [2,7,8,9,10,11], and general patients [10]. The cost of hemodialysis treatment has increased over time [12], even though hemodialysis patients have a poorer health-related quality of life (HRQOL), and kidney disease quality of life (KDQOL) than peritoneal dialysis patients [13]. Quality of life (QOL) is strongly associated with renal failure progression and death in CKD patients [14]. In addition, hemodialysis patients with a better HRQOL had a better adherence to recommendations [15]. However, hemodialysis patients commonly report a poor HRQOL [16,17]. QOL is under-investigated in CKD patients [18], especially in the Vietnamese context. 

Though there were a few studies that investigated the costs of dialysis treatments in low–middle income countries, there is a lack of studies conducted among hemodialysis patients in Vietnam [19,20]. In addition, it remains difficult for clinicians to provide individualized care based on socio-demographic characteristics as well as clinical and laboratory parameters [21]. It has been suggested that factors associated with a decline in HRQOL during renal progression should be investigated [22]. Therefore, we aim to investigate healthcare costs, HRQOL, KDQOL, and to explore their associated factors among ESRD patients treated in a hemodialysis center in central Vietnam.

## 2. Methods

### 2.1. Study Design and Settings

We conducted a clinical cross-sectional study from January to April 2019. Data were collected on ESRD patients receiving hemodialysis treatment at the Hemodialysis Department of Quang Ngai Provincial General Hospital (QNPGH). This is a level 2 hospital (lower than national hospitals at level 1), the highest level hospital in Quang Ngai province, and is located in central Vietnam. Its function and structure are similar to other level 2 provincial hospitals. Around 70% of hemodialysis patients in Quang Ngai province received treatments at QNPGH which follow the standard hemodialysis guidelines of the Ministry of Health in Vietnam [23].

### 2.2. Study Participants

Patients recruited were those aged 18 years or older, who received two or three hemodialysis sessions per week. Patients excluded were those with cognitive impairments, those receiving whole-day tube feeding, at the acute stage, hospitalized and those who had suffered a stroke. A sample of 160 out of 170 patients treated at the dialysis center was selected and analyzed. Ten stroke patients were excluded. Among the three main types of vascular access, including native arteriovenous fistula (AVF), arteriovenous graft (AVG), and vascular access catheter (VAC), the AVF was used for all studied patients in the current study. 

### 2.3. Instruments and Measurements

#### 2.3.1. Patient’s Characteristics

We assessed patients’ age (19–59, 60–83 years), gender (women vs. men), marital status (never married vs. ever married), educational attainment (illiterate/elementary vs. junior high school vs. senior high school or higher), occupation (no job vs. having a job). Residential areas were also assessed and classified into rural areas (countryside or remote areas) vs. urban areas (or metropolitan areas). Income was classified into two groups, e.g., below the poverty line (<VND 1.0 million/month for rural areas, <VND 1.3 million/month for urban areas), and above the poverty line (≥VND 1.0 million/month for rural areas, ≥VND 1.3 million/month for urban areas), with VND 1 x = VND 23,000 according to Vietcombank-State Bank of Vietnam [24].

#### 2.3.2. Clinical Parameters

Anthropometric parameters were measured, including waist circumference (WC, cm), height (cm), weight (kg). Body mass index (BMI) was calculated as weight (kg) divided by height (m)^2^. Overweight/obesity and abdominal obesity were defined as BMI ≥ 24.0 kg/m^2^) [25], and WC ≥ 90 for men or ≥80 for women, respectively [26]. Hemodialysis vintage (year) was recorded. Comorbidities were assessed using items of the Charlson comorbidity index [27,28]. Systolic blood pressure (SBP) or diastolic blood pressure (DBP) were also recorded. Hypertension was defined if SBP/DBP ≥ 140/90 mmHg [29,30,31]. 

#### 2.3.3. Biochemical Parameters

The laboratory data collected from medical records including red blood cells (RBC, 10^6^/μL), white blood cells (WBC, 10^3^/µL), hemoglobin (Hgb, g/dL), hematocrit (Hct, %) creatinine (Cre, mg/dL), pre-dialysis blood urea nitrogen (Pre-BUN, mg/dL); total protein (TP, mg/dL) and albumin (Alb, mg/dL). Anemia is defined as Hgb < 11 g/dL [32], or RBC < 4.2·10^6^/μL, or Hct < 40% [33]. Risk of malnutrition is defined as TP < 6 g/dL, Alb < 3.5 mg/dL, Cre < 7.5 mg/dL [34]. Moreover, pre-BUN was classified into two groups (pre-BUN < 20.0 mg/dL vs. pre-BUN ≥ 20.0 mg/dL), while WBC were also classified into two groups (WBC < 11.0·10^3^/µL vs. WBC ≥ 11.0·10^3^/µL).

#### 2.3.4. Healthcare Cost

All hemodialysis patients were covered by National Health Insurance (NHI). The total healthcare cost analyzed was the direct cost covered by NHI. In the current study, we analyzed monthly direct healthcare costs, e.g., costs of dialyzers and tubing, medical procedures, and costs related to laboratory testing and medications.

#### 2.3.5. Quality of Life

Quality of life (QOL) was assessed using the Kidney Disease Quality of Life Short Form (KDQOL-SF^TM^-v1.3) instrument, version 1.3 [35]. This questionnaire includes 36 items as the generic score (HRQOL), and 43 items as ESRD-targeted areas (KDQOL) [36]. The scores were calculated following the user manual [35,37]. The generic score of HRQOL is commonly used to assess the generic QOL of hemodialysis patients [38]. This has also been used among Vietnamese people [39,40]. The total score ranges from 0 (lowest) to 100 (highest), with higher scores indicating a better QOL. In the current study, we analyzed two scales in order to assess both overall and ESRD-specific aspects of QOL. HRQOL scale was assessed for the broad evaluation of general health across different domains, e.g., physical functioning, role physical, pain, general health, emotional wellbeing, role emotional, social function, and energy/fatigue. KDQOL scale was assessed for ESRD-specific QOL, e.g., symptoms/problems related to the scale of kidney disease, effects related to the scale of kidney disease, burden related to the scale of kidney disease, work status, cognitive function, quality of social interactions, sexual function, sleep, social support, dialysis staff encouragement, and patient satisfaction [36].

### 2.4. Data Collection Procedure

A doctor who worked at the dialysis center screened for qualified patients using the recruitment criteria. Researchers contacted eligible patients and asked for their voluntary participation. The informed consent form was signed by all participants prior to data collection. We conducted face-to-face interviews after the dialysis session. The printed questionnaire was used for assessing patients’ characteristics, and quality of life. Medical charts were used for assessing clinical and laboratory parameters. The expenditure was extracted from the hospital information system by a researcher.

### 2.5. Ethical Approval

This study was reviewed and approved by the Institutional Ethics Committee of Hue University of Medicine and Pharmacy, Vietnam (IRB No. H2018/218).

### 2.6. Statistical Analysis

Firstly, we used descriptive analysis to explore the studied variables’ distribution. A one-way ANOVA test was used to compare the distributions of healthcare cost, HRQOL, and KDQOL between the categories of socio-demographic factors and clinical and biochemical parameters. Secondly, bivariate and multivariate analyses were utilized to explore the associated factors of healthcare cost, HRQOL, and KDQOL. The factors analyzed in the multivariate model were those with significant associations with healthcare cost, HRQOL, and KDQOL in the bivariate model. To avoid multicollinearity, the correlations among socio-demographic factors, clinical, and biochemical parameters were estimated using Spearman’s correlation test. The representative factors were selected if moderate or high correlations existed. Data were analyzed using IBM SPSS version 20.0 (IBM Corp, Armonk, NY, USA). The *p*-value < 0.05 was set to define the significant level.

## 3. Results

### 3.1. Patients’ Characteristics

Out of the sample, 29.4% were aged 60 years or above, while 62.5% were men. The means of healthcare cost, HRQOL, and KDQOL were 9.4 ± 1.6, 45.1 ± 21.9, 51.3 ± 13.0, respectively. The distribution of healthcare cost varied by hemodialysis vintage, comorbidity, and albumin (*p* < 0.05). The distribution of HRQOL varied by age, residence area, comorbidity, albumin, and creatinine (*p* < 0.05). The distribution of KDQOL varied by age, education, residence area, occupation, comorbidity, hemoglobin, albumin, and creatinine (*p* < 0.05; Table 1). The proportions of RBC ≥ 4.2 (1.9%), Hct ≥ 40.0 (1.9%), WBC ≥ 11.0 (1.9%), and TP < 6.0 (5.6%) were relatively small. Therefore, we kept RBC, Hct, WBC, and TP as continuous variables for regression analysis.

### 3.2. Associated Factors of Healthcare Cost

In the bivariate analysis, the associated factors of healthcare cost were hemodialysis vintage, comorbidity, albumin, RBC and hematocrit (*p* < 0.05; Table 2). RBC and Hct were highly correlated (correlation coefficient, rho = 0.87; Table S1). Therefore, we kept age, gender, hemodialysis vintage, comorbidity, Alb, and Hct in the multivariate analysis. The results show that healthcare cost was significantly higher in patients with a dialysis vintage ≥ 5 years (regression coefficient (B): 0.74; 95% confidence interval (95% CI): 0.25, 1.23; *p* = 0.003) and with a comorbidity (B: 0.77; 95% CI: 0.24, 1.31; *p* = 0.005), compared to their counterparts. Patients with a higher hematocrit concentration had a lower healthcare cost (B: −0.07; 95% CI: −0.13, −0.01; *p* = 0.022; Table 2).

### 3.3. Associated Factors of HRQOL

In the bivariate analysis, associated factors of HRQOL were age, residence area, comorbidity, albumin and creatinine (*p* < 0.05; Table 3). Creatinine moderately correlated with albumin (rho = 0.43; Table S1). Therefore, we kept age, gender, residence area, comorbidity, and Alb in the multivariate analysis. The results show that the HRQOL score was statistically significantly higher in people who lived in an urban area (B: 9.08; 95% CI: 2.30, 15.85; *p* = 0.009) compared to those living in a rural area. Patients with a comorbidity (B: −14.20; 95% CI: −21.43, −6.97; *p* < 0.001) and with hypoalbuminemia (B: −9.31; 95% CI: −16.58, −2.04; *p* = 0.012) had lower HRQOL scores compared to their counterparts (Table 3). 

### 3.4. Associated Factors of KDQOL

In the bivariate analysis, associated factors of KDQOL were age, education, residence area, occupation, comorbidity, Hgb, Alb, and Cre. Since Cre correlated with Alb. Therefore, we kept age, gender, education, residence area, occupation, comorbidity, Hgb, and Alb in the multivariate model. The results show that KDQOL score was significantly higher in patients with higher educational levels (B: 5.38; 95% CI: 0.43, 10.34; *p* = 0.033 for junior high school; B: 6.29; 95% CI: 1.28, 11.30; *p* = 0.014 for senior high school or above) compared to those with education attainment at the illiterate or elementary school level. Patients with a comorbidity had lower KDQOL (B: −6.17; 95% CI: −10.49, −1.85; *p* = 0.005), compared to those without a comorbidity (Table 4).

## 4. Discussion

The results of our study show that higher education attainment was associated with better KDQOL. This is consistent with the findings of a previous study [41]. In addition, a marginal association between education and HRQOL was found in the bivariate analysis in our study. Previous studies showed that hemodialysis patients with higher education levels had a better HRQOL [41,42,43,44]. The establishment of an education and counseling system should be comprised of safe hemodialysis services to overcome the challenges of hemodialysis treatment in Vietnam [45,46].

In our study, people who resided in urban areas had a better HRQOL and a marginally better KDQOL compared to those who lived in rural areas. A previous study showed that chronic kidney disease patients who lived in rural areas had a poorer quality of life [41]. Similarly, people who lived in rural areas had more difficulties accessing and using healthcare services and experienced more health problems, and had a poorer HRQOL, as reported in a previous study in Vietnam [47].

We found that a longer hemodialysis vintage was associated with a higher healthcare cost. This can be explained by the fact that long-term dialysis patients possibly required more treatments, or experienced more complications [48,49,50,51], which, in turn, increased the treatment burden [52]. Comorbidities were associated with a higher healthcare cost in the current study. This is similar to the findings of a previous study that elucidated that patients with comorbid conditions (e.g., liver function disorder, hypertension, dyslipidemia, and cancer) spent more than those without dialysis treatment [53]. Moreover, patients with underlying health conditions had a poorer HRQOL and KDQOL, which we also found in our study. Previous studies reported that hemodialysis patients with less comorbid conditions had a better HRQOL [44,54,55], and a better KDQOL [44]. Another study showed that ESRD patients with a history of cardiovascular disease had a poorer HRQOL [43].

The association between hemoglobin and QOL was not found to be significant in our study and a previous one [54]. This finding is supported by a large trial on hemodialysis patients that reported no significant changes in quality of life between low and high Hgb groups or over time [56]. However, the results of another study showed that lower Hgb levels were associated with a poorer HRQOL in ESRD patients [43]. Additionally, our study showed that patients with a higher Hct concentration associated with a lower healthcare cost. Therefore, anemia is still an important issue in CKD patients and new treatment approaches are needed [57].

In the current study, malnutrition (hypoalbuminemia) was associated with poor HRQOL. This is similar to the findings of previous studies, which illustrated that higher albumin levels were associated with a better HRQOL [43,54]. In addition, poor nutritional status was found to be associated with higher healthcare expenditures, worse health outcomes [58], and a poorer HRQOL in hemodialysis patients [59].

In our study, the results show that socio-demographic factors (e.g., age, gender, marital status, occupation, and income) were not significantly associated with healthcare cost, HRQOL, and KDQOL. This was in line with a previous study, which illustrated that clinical parameters were better than socio-demographics at predicting HRQOL in ESRD patients [54]. Previous studies illustrated that age [60] and employment status [61] were significantly associated with HRQOL in CKD patients, and renal transplant recipients, respectively.

The current study had some limitations. Firstly, the study was conducted in a single hemodialysis center, with a relatively small sample size, which limits the generalizability of the findings. Secondly, we used a cross-sectional design to conduct the study, which cannot draw causality. There were potential biases which may have affected the results, e.g., reporting bias and the classification of residential areas (urban vs. rural). Therefore, the findings should be interpreted with caution. In addition, the adjusted R-square values for healthcare cost (0.151), HRQOL (0.170), and KDQOL (0.183) were relatively low. This suggests that a comprehensive assessment should be carried out in future studies to explore more associated factors, e.g., dialysis effectiveness indicators (Kt/V) and dietary intake. Despite these limitations, our findings raise important issues regarding healthcare cost and QOL in hemodialysis patients. Future studies should be conducted with larger samples in different settings, and with a longitudinal design, to examine the causal risk and protective factors of healthcare expenditure and QOL.

## 5. Conclusions

This is the first study that was conducted in Vietnam that aimed to explore the associated factors of healthcare cost, HRQOL, and KDQOL in hemodialysis patients. Patients with a longer hemodialysis vintage, a comorbidity, and a lower hematocrit concentration had a higher healthcare cost. Patients who lived in urban areas had a better HRQOL, while those with a higher education level had a better KDQOL. Patients with a comorbidity had a lower HRQOL and KDQOL. Malnourished patients had a lower HRQOL. Effective interventions are required to reduce healthcare costs and improve QOL in both aspects (general and ESRD-specific) and to focus on those hemodialysis patients with underlying health conditions such as anemia and malnutrition, those who reside in rural areas, and those with a lower educational attainment.

## Figures and Tables

**Table 1:** Participants’ characteristics, healthcare cost, health- and renal-related quality of life (*N* = 160).

Variables	Total Sample	Healthcare Cost *		HRQOL		KDQOL	
	*n* (%)	Mean ± SD	*p* **	Mean ± SD	*p* **	Mean ± SD	*p* **
**Characteristics**							
Age, year			0.059		0.030		0.001
19–59	113 (70.6)	9.6 ± 1.6		47.5 ± 21.1		53.4 ± 13.1	
60–83	47 (29.4)	9.1 ± 1.5		39.2 ± 22.9		46.3 ± 11.4	
Gender			0.732		0.491		0.847
Women	60 (37.5)	9.4 ± 1.7		46.6 ± 22.8		51.5 ± 12.3	
Men	100 (62.5)	9.4 ± 1.5		44.1 ± 21.4		51.1 ± 13.5	
Marital status			0.371		0.532		0.127
Never married	25 (15.6)	9.7 ± 1.7		47.6 ± 17.0		54.9 ± 10.6	
Ever married	135 (84.4)	9.4 ± 1.5		44.6 ± 22.7		50.6 ± 13.3	
Education			0.416		0.130		0.001
Illiterate or elementary	54 (33.8)	9.2 ± 1.7		40.2 ± 22.4		45.8 ± 11.0	
Junior high school	52 (32.6)	9.5 ± 1.6		47.7 ± 21.4		52.9 ± 12.6	
Senior high school or above	54 (33.8)	9.5 ± 1.4		47.4 ± 21.4		55.3 ± 13.6	
Residence area			0.740		0.029		0.034
Rural	109 (68.1)	9.4 ± 1.5		42.5 ± 21.6		49.8 ± 12.4	
Urban	51 (31.9)	9.5 ± 1.7		50.6 ± 21.7		54.5 ± 13.7	
Occupation			0.376		0.238		0.023
No job	120 (75.0)	9.3 ± 1.6		43.9 ± 21.1		49.9 ± 12.5	
Having job	40 (25.0)	9.6 ± 1.4		48.6 ± 24		55.3 ± 13.9	
Income ***			0.335		0.466		0.805
Below poverty line	119 (74.4)	9.3 ± 1.5		45.8 ± 20.6		51.4 ± 12.3	
Above poverty line	41 (25.6)	9.6 ± 1.9		42.9 ± 25.5		50.8 ± 14.9	
**Clinical parameters**							
BMI, kg/m^2^			0.954		0.250		0.163
BMI < 24.0	145 (90.6)	9.4 ± 1.6		44.4 ± 21.6		50.8 ± 13.0	
BMI ≥ 24.0	15 (9.4)	9.4 ± 1.2		51.3 ± 24.3		55.8 ± 12.2	
WC ****, cm			0.253		0.136		0.103
Normal	121 (75.6)	9.5 ± 1.6		43.6 ± 21.5		50.3 ± 13.8	
Obese	39 (24.4)	9.2 ± 1.4		49.6 ± 22.9		54.2 ± 9.6	
HD vintage, year			0.009		0.652		0.919
<5	64 (40)	9.0 ± 1.8		46.0 ± 22.8		51.2 ± 12.7	
≥5	96 (60)	9.7 ± 1.3		44.4 ± 21.4		51.4 ± 13.3	
Comorbidity			0.001		< 0.001		0.002
None	116 (72.5)	9.2 ± 1.5		49.4 ± 20.5		53.2 ± 12.8	
One or more	44 (27.5)	10.1 ± 1.6		33.5 ± 21.4		46.1 ± 12.3	
SBP, mmHg			0.420		0.145		0.177
SBP < 140	14 (8.8)	9.1 ± 1.1		36.9 ± 19.9		46.8 ± 13.8	
SBP ≥ 140	146 (91.3)	9.4 ± 1.6		45.8 ± 22.0		51.7 ± 12.9	
DBP, mmHg			0.357		0.937		0.560
DBP < 90	55 (34.4)	9.3 ± 1.6		44.9 ± 20.8		50.5 ± 12.3	
DBP ≥ 90	105 (65.6)	9.5 ± 1.6		45.2 ± 22.6		51.7 ± 13.4	
**Laboratory parameters**							
Hgb, g/dL			0.366		0.097		0.035
Hgb ≥ 11.0	26 (16.3)	9.2 ± 0.8		38.5 ± 22.7		46.4 ± 13.1	
Hgb < 11.0	134 (83.8)	9.5 ± 1.7		46.3 ± 21.6		52.2 ± 12.8	
Alb, mg/dL			0.028		0.002		0.011
Alb ≥ 3.5	117 (73.1)	9.2 ± 1.4		48.2 ± 21.9		52.9 ± 12.7	
Alb < 3.5	43 (26.9)	9.9 ± 1.8		36.4 ± 19.8		47.0 ± 12.9	
Cre, mg/dL			0.804		<0.001		<0.001
Cre ≥ 7.5	135 (84.4)	9.4 ± 1.5		47.9 ± 21.1		52.9 ± 12.6	
Cre < 7.5	25 (15.6)	9.5 ± 1.8		29.7 ± 20.1		42.4 ± 11.7	
Pre-BUN, mg/dL			0.640		0.083		0.147
Pre-BUN < 20.0	32 (20.0)	9.5 ± 1.7		39.1 ± 22.1		48.3 ± 13.1	
Pre-BUN ≥ 20.0	128 (80.0)	9.4 ± 1.5		46.6 ± 21.7		52.0 ± 12.9	
RBC, 10^6^/μL			0.942		0.796		0.467
RBC ≥ 4.2	3 (1.9)	9.5 ± 0.5		41.8 ± 12.2		45.9 ± 6.1	
RBC < 4.2	157 (98.1)	9.4 ± 1.6		45.1 ± 22.1		51.4 ± 13.1	
Hct, %			0.437		0.921		0.704
Hct ≥ 40.0	3 (1.9)	8.7 ± 1.0		43.8 ± 9.3		48.4 ± 1.6	
Hct < 40.0	157 (98.1)	9.4 ± 1.6		45.1 ± 22.1		51.3 ± 13.1	
WBC, 10^3^/µL			0.325		0.422		0.336
WBC < 11.0	157 (98.1)	9.4 ± 1.6		45.3 ± 21.9		51.4 ± 13.0	
WBC ≥ 11.0	3 (1.9)	10.3 ± 2.2		35.0 ± 21.5		44.1 ± 9.7	
TP, g/dL			0.088		0.622		0.281
TP ≥ 6.0	151 (94.4)	9.4 ± 1.5		44.8 ± 22.0		51.6 ± 13.0	
TP < 6.0	9 (5.6)	10.3 ± 2.0		48.6 ± 20.7		46.7 ± 12.2	
Healthcare cost *, mean ± SD	9.4 ± 1.6						
HRQOL, Mean ± SD	45.1 ± 21.9						
KDQOL, Mean ± SD	51.3 ± 13.0						

Abbreviations: HRQOL, health-related quality of life; KDQOL, kidney disease quality of life; SD, standard deviation; BMI, body mass index; WC, waist circumference; HD, hemodialysis; SBP, systolic blood pressure; DBP, diastolic blood pressure; Hgb, hemoglobin; Alb, albumin; Cre, creatinine; pre-BUN, pre-dialysis blood urea nitrogen; RBC, red blood cells; Hct, hematocrit; WBC, white blood cells; TP, total protein. *: The total healthcare cost was measure in millions of VND (USD 1 = VND 23,000 according to Vietcombank—the state bank of Vietnam). **: Result of ANOVA test. ***: Monthly income was classified into below the poverty line (<VND 1.0 million/month for rural areas, <VND 1.3 million/month for urban areas), and above the poverty line (≥VND 1.0 million/month for rural areas, ≥VND 1.3 million/month for urban areas). ****: Abdominal obesity was defined as WC ≥ 90 for men or ≥80 for women, respectively.

**Table 2:** Associated factors of healthcare cost via bivariate and multivariate linear regression analyses (*N* = 160).

Variables	Healthcare Cost*			
	Bivariate		Multivariate	
	B (95% CI)	*p*	B (95% CI)	*p*
**Characteristics**				
Age, year				
19–59				
60–83	−0.51 (−1.05, 0.02)	0.059	−0.49 (−1.00, 0.03)	0.067
Gender				
Women				
Men	0.09 (−0.42, 0.60)	0.732	0.132 (−0.35, 0.61)	0.587
Marital status				
Never married				
Ever married	−0.31 (−0.98, 0.37)	0.371		
Education				
Illiterate or elementary				
Junior high school	0.34 (−0.26, 0.94)	0.269		
Senior high school or above	0.36 (−0.24, 0.95)	0.238		
Residence area				
Rural				
Urban	0.09 (−0.44, 0.62)	0.740		
Occupation				
No job				
Having job	0.25 (−0.31, 0.82)	0.376		
Income **				
Below poverty				
Above poverty	0.27 (−0.29, 0.84)	0.335		
**Clinical parameters**				
BMI, kg/m^2^				
BMI < 24.0				
BMI ≥ 24.0	−0.02 (−0.87, 0.82)	0.954		
WC ***, cm				
Normal				
Obese	−0.33 (−0.90, 0.24)	0.253		
HD vintage, year				
<5				
≥5	0.66 (0.17, 1.15)	0.009	0.74 (0.25, 1.23)	0.003
Comorbidity				
None				
One or more	0.89 (0.36, 1.43)	0.001	0.77 (0.24, 1.31)	0.005
SBP, mmHg				
SBP < 140				
SBP ≥ 140	0.36 (−0.51, 1.22)	0.420		
DBP, mmHg				
DBP < 90				
DBP ≥ 90	0.24 (−0.27, 0.76)	0.357		
**Laboratory parameters**				
Hgb, g/dL				
Hgb ≥ 11.0				
Hgb < 11.0	0.31 (−0.36, 0.97)	0.366		
Alb, mg/dL				
Alb ≥ 3.5				
Alb < 3.5	0.61 (0.07, 1.16)	0.028	0.28 (−0.27, 0.83)	0.312
Cre, mg/dL				
Cre ≥ 7.5				
Cre < 7.5	0.09 (−0.59, 0.76)	0.804		
Pre-BUN, mg/dL				
Pre-BUN < 20				
Pre-BUN ≥ 20	−0.15 (−0.76, 0.47)	0.640		
RBC, 10^6^/μL	−0.55 (−1.02, −0.09)	0.021		
Hct, %	−0.08 (−0.13, −0.02)	0.005	−0.07 (−0.13, −0.01)	0.022
WBC, 10^3^/µL	0.01 (−0.12, 0.14)	0.919		
TP, mg/dL	0.05 (−0.38, 0.48)	0.814		
Adjusted R square			0.151	

Abbreviations: B, regression coefficient; CI, confidence interval; BMI, body mass index; WC, waist circumference; HD, hemodialysis; SBP, systolic blood pressure; DBP, diastolic blood pressure; Hgb, hemoglobin; Alb, albumin; Cre, creatinine; pre-BUN, pre-dialysis blood urea nitrogen; RBC, red blood cells; Hct, hematocrit; WBC, white blood cells; TP, total protein. *: The total healthcare cost was measure in millions of VND (USD 1 = VND 23,000 according to Vietcombank-State Bank of Vietnam). **: Monthly income was classified into below the poverty line (<VND 1.0 million/month for rural areas, < VND 1.3 million/month for urban areas), and above the poverty line (≥VND 1.0 million/month for rural areas, ≥VND 1.3 million/month for urban areas). ***: Abdominal obesity was defined as WC ≥ 90 for men or ≥80 for women, respectively.

**Table 3:** Associated factors of health-related quality of life via bivariate and multivariate linear regression analyses (*N* = 160).

Variables	HRQOL			
	Bivariate		Multivariate	
	B (95%CI)	*p*	B (95%CI)	*p*
**Characteristics**				
Age, year				
19–59				
60–83	−8.25 (−15.68, −0.83)	0.030	−5.89 (−12.83, 1.05)	0.096
Gender				
Women				
Men	−2.48 (−9.56, 4.60)	0.491	−1.80 (−8.42, 4.83)	0.593
Marital status				
Never married				
Ever married	−2.99 (−12.44, 6.45)	0.532		
Education				
Illiterate or elementary				
Junior high school	7.54 (−0.81, 15.90)	0.076		
Senior high school or above	7.26 (−1.02, 15.53)	0.085		
Residence area				
Rural				
Urban	8.08 (0.82, 15.33)	0.029	9.08 (2.30, 15.85)	0.009
Occupation				
No job				
Having job	4.73 (−3.16, 12.63)	0.238		
Income *				
Below poverty				
Above poverty	−2.91 (−10.76, 4.94)	0.466		
**Clinical parameters**				
BMI, kg/m^2^				
BMI < 24.0				
BMI ≥ 24.0	6.85 (−4.87, 18.58)	0.250		
WC **, cm				
Normal				
Obese	6.02 (−1.92, 13.96)	0.136		
HD vintage, year				
<5				
≥5	−1.60 (−8.60, 5.40)	0.652		
Comorbidity				
None				
One or more	−15.95 (−23.21, −8.68)	<0.001	−14.20 (−21.43, −6.97)	<0.001
SBP, mmHg				
SBP < 140				
SBP ≥ 140	8.94 (−3.12, 21.01)	0.145		
DBP, mmHg				
DBP < 90				
DBP ≥ 90	0.29 (−6.94, 7.52)	0.937		
**Laboratory parameters**				
Hgb, g/dL				
Hgb ≥ 11.0				
Hgb < 11.0	7.80 (−1.43, 17.02)	0.097		
Alb, mg/dL				
Alb ≥ 3.5				
Alb < 3.5	−11.77 (−19.29, −4.25)	0.002	−9.31 (−16.58, −2.04)	0.012
Cre, mg/dL				
Cre ≥ 7.5				
Cre < 7.5	−18.22 (−27.23, −9.21)	<0.001		
Pre-BUN, mg/dL				
Pre-BUN < 20				
Pre-BUN ≥ 20	7.50 (−1.00, 16.00)	0.083		
RBC, 10^6^/μL	−0.34 (−6.98, 6.30)	0.919		
Hct, %	−0.20 (−0.98, 0.58)	0.613		
WBC, 10^3^/µL	−1.01 (−2.85, 0.82)	0.277		
TP, mg/dL	−1.19 (−7.14, 4.76)	0.694		
Adjusted R square			0.170	

Abbreviations: HRQOL, health-related quality of life; B, regression coefficient; CI, confidence interval; BMI, body mass index; WC, waist circumference; HD, hemodialysis; SBP, systolic blood pressure; DBP, diastolic blood pressure; Hgb, hemoglobin; Alb, albumin; Cre, creatinine; pre-BUN, pre-dialysis blood urea nitrogen; RBC, red blood cells; Hct, hematocrit; WBC, white blood cells; TP, total protein. *: Monthly income was classified into below the poverty line (<VND 1.0 million/month for rural areas, <VND 1.3 million/month for urban areas), and above the poverty line (≥VND 1.0 million/month for rural areas, ≥VND 1.3 million/month for urban areas). **: Abdominal obesity was defined as WC ≥ 90 for men or ≥80 for women, respectively.

**Table 4:** Associated factors of kidney disease quality of life via bivariate and multivariate linear regression analyses (*N* = 160).

Variables	KDQOL			
	Bivariate		Multivariate	
	B (95% CI)	*p*	B (95% CI)	*p*
**Characteristics**				
Age, year				
19–59				
60–83	−7.13 (−11.46, −2.80)	0.001	−3.66 (−8.08, 0.77)	0.104
Gender				
Women				
Men	−0.41 (−4.62, 3.80)	0.847	−0.25 (−4.25, 3.76)	0.904
Marital status				
Never married				
Ever married	−4.32 (−9.89, 1.25)	0.127		
Education				
Illiterate or elementary				
Junior high school	7.12 (2.35, 11.89)	0.004	5.38 (0.43, 10.34)	0.033
Senior high school or above	9.49 (4.76, 14.21)	< 0.001	6.29 (1.28, 11.30)	0.014
Residence area				
Rural				
Urban	4.67 (0.36, 8.99)	0.034	4.02 (−0.08, 8.11)	0.055
Occupation				
No job				
Having job	5.39 (0.76, 10.02)	0.023	1.96 (−2.59, 6.51)	0.396
Income *				
Below poverty				
Above poverty	−0.59 (−5.25, 4.08)	0.805		
**Clinical parameters**				
BMI, kg/m^2^				
BMI < 24.0				
BMI ≥ 24.0	4.93 (−2.01, 11.88)	0.163		
WC **, cm				
Normal				
Obese	3.90 (−0.8, 8.61)	0.103		
HD vintage, year				
<5				
≥5	0.22 (−3.94, 4.37)	0.919		
Comorbidity				
None				
One or more	−7.12 (−11.54, −2.69)	0.002	−6.17 (−10.49, −1.85)	0.005
SBP, mmHg				
SBP < 140				
SBP ≥ 140	4.92 (−2.25, 12.09)	0.177		
DBP, mmHg				
DBP < 90				
DBP ≥ 90	1.27 (−3.02, 5.55)	0.560		
**Laboratory parameters**				
Hgb, g/dL				
Hgb ≥ 11.0				
Hgb < 11.0	5.88 (0.43, 11.32)	0.035	4.23 (−0.98, 9.43)	0.111
Alb, mg/dL				
Alb ≥ 3.5				
Alb < 3.5	−5.88 (−10.38, −1.38)	0.011	−3.17 (−7.55, 1.21)	0.155
Cre, mg/dL				
Cre ≥ 7.5				
Cre < 7.5	−10.48 (−15.84, −5.11)	< 0.001		
Pre-BUN, mg/dL				
Pre-BUN < 20				
Pre-BUN ≥ 20	3.73 (−1.33, 8.79)	0.147		
RBC, 10^6^/μL	−1.22 (−5.15, 2.72)	0.543		
Hct, %	−0.15 (−0.61, 0.31)	0.519		
WBC, 10^3^/µL	−0.61 (−1.70, 0.48)	0.270		
TP, mg/dL	−0.50 (−4.03, 3.03)	0.781		
Adjusted R square			0.183	

Abbreviations: KDQOL, kidney disease quality of life; B, regression coefficient; CI, confidence interval; BMI, body mass index; WC, waist circumference; HD, hemodialysis; SBP, systolic blood pressure; DBP, diastolic blood pressure; Hgb, hemoglobin; Alb, albumin; Cre, creatinine; pre-BUN, pre-dialysis blood urea nitrogen; RBC, red blood cells; Hct, hematocrit; WBC, white blood cells; TP, total protein. *: Monthly income was classified into below the poverty line (<1.0 million VND/month for rural areas, <1.3 million VND/month for urban areas), and above the poverty line (≥1.0 million VND/month for rural areas, ≥1.3 million VND/month for urban areas). **: Abdominal obesity was defined as WC ≥ 90 for men or ≥80 for women, respectively.

## Supplementary Materials

The following are available online at https://www.mdpi.com/1660-4601/17/18/6552/s1.
