# Supplementary material for: Associations of Socio-Demographic, Clinical and Biochemical Parameters with Healthcare Cost, Health- and Renal-Related Quality of Life in Hemodialysis Patients: A Clinical Observational Study
Source: Int J Environ Res Public Health. 2020 Sep 9;17(18):6552. doi: 10.3390/ijerph17186552 (PMC7559218; doi:10.3390/ijerph17186552)
Supplement: Supplementary file 1 [file ijerph-17-06552-s001.pdf]

International Journal of
Environmental Research and Public Health

MDPI

Table 1. Spearman's correlation among independent variables (N = 160).

	Age	Gender	Marital status	Education	Residence area	Occupation	Income	BMI	WC	HD vintage	CCI	SBP	DBP	Hgb	Alb	Cre	Pre-BUN	RBC	Hct	WBC	TP
Age	1.00																				
Gender	0.05	1.00																			
Marital status	0.20	0.09	1.00																		
Education	−0.22	0.11	−0.17	1.00																	
Residence area	0.03	−0.11	−0.04	0.20	1.00																
Occupation	−0.06	0.00	0.05	0.30	0.10	1.00															
Income	0.09	0.07	0.09	0.30	0.12	**0.45**	1.00														
BMI	0.06	0.04	−0.03	0.22	0.21	0.15	0.14	1.00													
WC	0.08	−0.37	−0.04	0.04	0.27	0.08	0.13	**0.33**	1.00												
HD vintage	−0.09	0.05	−0.04	0.16	−0.04	0.06	−0.02	−0.05	0.02	1.00											
CCI	0.16	0.07	0.03	−0.02	0.09	−0.10	0.09	−0.10	−0.06	−0.01	1.00										
SBP	−0.04	0.03	0.05	−0.05	−0.07	−0.03	−0.02	−0.01	0.02	0.02	0.04	1.00									
DBP	−0.05	0.09	0.05	0.08	0.02	0.05	0.06	0.08	−0.08	0.03	0.06	0.24	1.00								
Hgb	0.14	0.14	0.10	0.09	0.00	−0.09	0.02	0.12	0.12	0.21	−0.18	−0.13	−0.09	1.00							
Alb	−0.11	0.16	0.07	0.15	−0.07	0.14	0.01	0.09	−0.07	−0.03	−0.22	0.07	0.06	0.26	1.00						
Cre	−0.17	**0.33**	0.03	0.14	−0.02	0.02	−0.01	0.15	0.02	−0.12	−0.22	0.15	0.06	0.06	**0.43**	1.00					
Pre−BUN	0.09	0.03	0.08	−0.01	0.06	0.04	−0.16	0.08	−0.02	−0.20	−0.03	0.04	0.02	−0.18	0.15	**0.42**	1.00				
RBC	0.04	0.08	0.05	0.02	−0.11	−0.11	−0.04	0.04	0.06	0.06	−0.25	−0.16	−0.10	**0.79**	0.29	0.13	−0.16	1.00			
Hct	0.14	0.09	0.08	0.08	−0.02	−0.12	−0.01	0.12	0.11	0.19	−0.20	−0.14	−0.10	**0.95**	0.23	0.04	−0.20	**0.87**	1.00		
WBC	0.05	0.04	0.11	−0.11	0.00	−0.07	−0.01	0.10	0.11	−0.09	−0.02	−0.04	0.19	0.12	0.15	0.21	−0.05	0.14	0.10	1.00	
TP	−0.02	−0.01	0.02	−0.05	−0.07	0.11	0.02	0.05	0.01	0.01	−0.08	−0.01	0.08	0.21	**0.41**	0.10	0.01	0.21	0.19	0.19	1.00

Abbreviations: BMI, body mass index; WC, waist circumference; HD, hemodialysis; CCI, Charlson comorbidity index; SBP, systolic blood pressure; DBP, diastolic blood pressure; Hgb, hemoglobin; Alb, albumin; Cre, creatinine; pre-BUN, pre-dialysis blood urea nitrogen; RBC, red blood cell; Hct, hematocrit; WBC, white blood cell; TP, total protein.
